# New York Journal of Medicine

**Published:** 1856-04

**Authors:** 


*New York Journal of Medicine.*—The January No. of this old periodical contains an admirable portrait of the late Dr. T. Romeyn Beck, of Albany, with a biographical memoir; and its usual amount of original and selected matter superadded. Nearly thirty pages are occupied by Dr. Markoe, of the N. Y. Hospital, in advocacy of amputation at the knee joint, instead of the thigh, as less liable to prove fatal. The points he attempts to make are thus summed up, viz:—*American Med. Gaz.*

In recapitulation of the points advanced in this paper, it would appear

1. That the amputation at the knee joint has been regarded with disfavor and rejected by the mass of the profession.

2. The results of experience seem to warrant and demand a reconsideration of this unfavorable verdict.

3. It is found that the stump left after this operation is much more useful than that left after amputation of the thigh, it being capable of bearing well and easily the weight of the body on an artificial limb—a consideration involving great comfort to the rich man, who wears an artificial limb; and on the poor man, who must otherwise walk with a crutch, conferring the power of walking on an ordinary peg leg, thereby enabling him to earn his livelihood.

4. This amputation is, probably, attended with less constitutional shock, inasmuch as it is performed farther from the trunk than amputation through the thigh.

5. The wound made in section of knee joint is really less extensive than in amputation higher up.

6. No muscular interspaces are exposed by the knife, and, therefore, there is less danger of inflammation spreading up the thigh.

7. Fewer ligatures, and those more favorably situated, are used in this operation.

8. The muscles moving the stump are not severed, and the patient is able, therefore, to move the stump with extraordinary facility, and is but little liable to painful muscular twitchings.

9. No retraction of muscles can exist, by which the coverings are drawn back from the end of the bone, producing conical stump.

10. The bone is unwounded in this amputation, and thus the numerous and serious dangers which arise from sawing the bone, and exposing its medullary cavity to air and pus, cannot exist.

11. The dangers apprehended from opening so large an articulation have been very greatly exaggerated, and are, in practice, found to be of rare occurrence.

12. Statistics show that the mortality, after this operation, compares favorably with amputation of the thigh, being 37 per cent. of deaths in the former, against 43½ per cent. in the latter.

13. The best mode of operation, when we have a choice, is by the long anterior, and short posterior, flap.

14. The verdict of some of the first French and English surgeons, is decidedly in favor of the knee joint operation.

